# Frontotemporal dementia–amyotrophic lateral sclerosis syndrome locus on chromosome 16p12.1–q12.2: genetic, clinical and neuropathological analysis

**DOI:** 10.1007/s00401-013-1078-9

**Published:** 2013-01-22

**Authors:** Carol Dobson-Stone, Agnes A. Luty, Elizabeth M. Thompson, Peter Blumbergs, William S. Brooks, Cathy L. Short, Colin D. Field, Peter K. Panegyres, Jane Hecker, Jennifer A. Solski, Ian P. Blair, Janice M. Fullerton, Glenda M. Halliday, Peter R. Schofield, John B. J. Kwok

**Affiliations:** 1 Neuroscience Research Australia, Barker St, Randwick, Sydney, NSW 2031 Australia; 2 University of New South Wales, Sydney, Australia; 3 SA Clinical Genetics Service, SA Pathology, Women’s and Children’s Hospital, Adelaide, Australia; 4 Department of Paediatrics, University of Adelaide, North Terrace, Adelaide, Australia; 5 Institute of Medical and Veterinary Science, Adelaide, Australia; 6 Department of Neurology, The Queen Elizabeth Hospital, Woodville, Adelaide, Australia; 7 Division of Rehabilitation and Aged Care, Memory Clinic, Repatriation General Hospital, Daw Park, Adelaide, Australia; 8 Present Address: Adelaide Dementia Driving Clinic, North Adelaide, Australia; 9 Neurodegenerative Disorders Research Pty Ltd, Subiaco, Perth, Australia; 10 Department of General Medicine, Royal Adelaide Hospital, Adelaide, Australia; 11 Northcott Neuroscience Laboratory, ANZAC Research Institute, Concord Hospital, Sydney, Australia

**Keywords:** Frontotemporal dementia, Amyotrophic lateral sclerosis, Motor neuron disease, Corticobasal degeneration, Tau, TDP-43

## Abstract

Numerous families exhibiting both frontotemporal dementia (FTD) and amyotrophic lateral sclerosis (ALS) have been described, and although many of these have been shown to harbour a repeat expansion in *C9ORF72*, several *C9ORF72*-negative FTD-ALS families remain. We performed neuropathological and genetic analysis of a large European Australian kindred (Aus-12) with autosomal dominant inheritance of dementia and/or ALS. Affected Aus-12 members developed either ALS or dementia; some of those with dementia also had ALS and/or extrapyramidal features. Neuropathology was most consistent with frontotemporal lobar degeneration with type B TDP pathology, but with additional phosphorylated tau pathology consistent with corticobasal degeneration. Aus-12 DNA samples were negative for mutations in all known dementia and ALS genes, including *C9ORF72* and *FUS*. Genome-wide linkage analysis provided highly suggestive evidence (maximum multipoint LOD score of 2.9) of a locus on chromosome 16p12.1–16q12.2. Affected individuals shared a chromosome 16 haplotype flanked by D16S3103 and D16S489, spanning 37.9 Mb, with a smaller suggestive disease haplotype spanning 24.4 Mb defined by recombination in an elderly unaffected individual. Importantly, this smaller region does not overlap with *FUS*. Whole-exome sequencing identified four variants present in the maximal critical region that segregate with disease. Linkage analysis incorporating these variants generated a maximum multipoint LOD score of 3.0. These results support the identification of a locus on chromosome 16p12.1–16q12.2 responsible for an unusual cluster of neurodegenerative phenotypes. This region overlaps with a separate locus on 16q12.1–q12.2 reported in an independent ALS family, indicating that this region may harbour a second major locus for FTD-ALS.

**Electronic supplementary material:**

The online version of this article (doi:10.1007/s00401-013-1078-9) contains supplementary material, which is available to authorized users.

## Introduction

Frontotemporal dementia (FTD) is a clinically and pathologically heterogeneous group of disorders that can present with personality and behavioural changes or language deficits [33]. The discovery of *MAPT* mutations in families with hereditary dementia with parkinsonism [16] allowed the unified classification of a group of families with a wide spectrum of clinical and pathological diagnoses. Dementia in these families was often atypical but most resembled FTD [27], with changes in personality, behaviour and insight, together with extrapyramidal motor features. Similarly, a group of *MAPT*-negative families with heterogeneous clinical features was later shown to have mutations in the granulin gene (*GRN*) [6]. Some families with *GRN* mutations have a diagnosis of corticobasal syndrome (CBS) [7, 30].

Amyotrophic lateral sclerosis (ALS) is characterised by degeneration of upper and lower motor neurons, leading to progressive muscle wasting, spasticity and ultimately paralysis and death. Families with hereditary dementia and ALS have been linked to a *C9ORF72* hexanucleotide repeat expansion [9, 31]. These families usually include some affected members with pure ALS, while the majority of affected members develop a dementia syndrome that is often atypical or difficult to diagnose, but in most cases eventually resembles FTD. Affected members with dementia may also develop ALS during the course of their disease. As well as *MAPT*, *GRN* and *C9ORF72*, other genes have been found to harbour mutations in families with FTD (e.g., *VCP*, *CHMP2B*) or ALS (e.g., *SOD1*, *TARDBP*, *FUS*) [3, 35].

Several types of neuropathology are found in patients with FTD, classified under the general rubric of frontotemporal lobar degeneration (FTLD). Most cases contain filamentous inclusions made of one of three constitutive neuronal proteins: tau (FTLD-tau), TAR DNA-binding protein (FTLD-TDP) or fused in sarcoma (FTLD-FUS) [22]. Cases with FTD-ALS most commonly have type B FTLD-TDP pathology characterised by motor neuron-like neuronal cytoplasmic inclusions (NCIs) [32]. In contrast, corticobasal degeneration (CBD) has a distinctive FTLD-tau pathology with ballooned neurons and tau-immunoreactive astrocytic plaques and threads [10]. In this study, we describe a large multigenerational family (Aus-12) with a clinical spectrum of FTD and ALS, and neuropathology consistent with CBD FTLD-tau and type B FTLD-TDP. Combined genome-wide linkage and whole-exome sequencing analysis identified a disease locus on chromosome 16p12.1–16q12.2.

## Subjects and methods

### Family recruitment

The proband (IV:23) was referred to a memory clinic at age 56 with symptoms suggesting cognitive decline in the context of a family history of young-onset dementia. She subsequently visited a clinical genetics service, where a detailed family history was compiled including available medical records. Twelve individuals with dementia and two with ALS were identified over four generations in a pattern consistent with autosomal dominant inheritance. Approval to approach relatives for a family genetics study was granted by the Ethics Committee of the Women’s and Children’s Hospital, Adelaide, and genetic studies were approved by the Ethics Committee of Concord Hospital in Sydney. The next of kin of individuals IV:5 and IV:7 consented to an autopsy study for brain research at the time of death and tissue sections and neuropathological reports for this study were obtained from the South Australian Brain Bank. To determine final diagnoses for IV:5 and IV:7, a retrospective review of their neuropathology was performed using current diagnostic criteria for Alzheimer’s disease, dementia with Lewy bodies, FTLD, ALS, and other neurodegenerative syndromes including CBD, progressive supranuclear palsy, and vascular dementia [8, 10, 15, 17, 22, 24, 26]. Brain tissue from a previously published familial FTLD-TDP case with a R493X *GRN* mutation [29] and two neuropathologically confirmed CBD FTLD-tau cases, obtained from the Sydney Brain Bank, were examined for comparison.

### Immunohistochemical analysis

Immunohistochemistry was performed on formalin-fixed paraffin-embedded 7–10-μm superior frontal cortex, hippocampus or spinal cord sections. We performed standard peroxidase immunohistochemistry with citrate buffer antigen retrieval and 0.5 % cresyl violet counterstaining [19]. Antibodies used were for ubiquitin (Z0458, DAKO, Denmark, diluted 1:200), phosphorylated tau (MN1020, PIERCE, USA, diluted 1:10,000), phosphorylated TDP (BC001487, PTG, USA, diluted 1:500), FUS (HPA008784, Sigma, Australia, diluted 1:1,000), α-internexin (32-3600, ZYMED Laboratories, USA, diluted 1:50) and phosphorylated 200 kD neurofilament (MAS330, Seralab, UK, diluted 1:200). Reaction specificity was confirmed by omitting primary antibodies. Double immunofluorescent labelling was performed to detect phosphorylated tau and phosphorylated TDP. After antigen retrieval, sections were treated for autofluorescence by immersion in 0.25 % potassium permanganate solution for 7 min, rinsed with water and placed in 1 % potassium metabisulphite, 1 % oxalic acid until the sections returned to their original colour. Tau and TDP immunoreactivity was visualised with Alexa Fluor 568 goat anti-rabbit and Alexa Fluor 488 goat anti-mouse secondary antibodies (Invitrogen) on a confocal microscope (C190; Nikon Corporation, Tokyo, Japan). A section without primary antibodies was included for each staining procedure as a negative control. In addition, a mixture of the secondary antibodies was applied to sections with only one primary antibody incubated on each section.

FTLD-TDP was classified into one of four subtypes [21] based on the morphology and laminar distribution of TDP-immunopositive inclusions in the affected brain regions.

### Genetic evaluation of family

Blood was collected from 13 family members and DNA extracted. DNA was screened for mutations in known dementia/ALS genes (Supplementary Table 1). A 10-cM genome-wide scan was performed on DNA from 12 individuals by the Australian Genome Research Facility (AGRF) with microsatellite markers from the ABI-400 set (ABI Prism Linkage Mapping Set, version 2.5, MD-10). Parametric pair-wise and multipoint LOD scores were calculated and simulation analyses were performed using MERLIN v1.1.2 [2]. Autosomal dominant inheritance of a single genetic locus for all clinical variants was assumed with a phenocopy rate of 0.005, a disease gene frequency of 0.001 and equal marker allele frequencies. Seven liability classes were established based on pedigree data with 1 % penetrance—age < 25 years, 8 %—between 26 and 34 years, 22 %—between 35 and 44 years, 46 %—between 45 and 54 years, 71 %—between 55 and 64 years, 91 %—between 65 and 74 years, and 95 %—age > 75 years. Individuals were assigned a liability class based on age of onset for affected cases and age at last consultation for asymptomatic cases.

High-resolution fine mapping of chromosome 16 was performed at the AGRF using microsatellite markers selected from the UCSC Human Genome Browser Gateway (http://genome.ucsc.edu/cgi-bin/hgGateway). Primers were fluorescently labelled with 6-FAM and PCR was carried out according to standard protocols. For fine mapping, allele frequencies were derived from a cohort of European ancestry Australian normals [13], from CEPH family data (http://www.cephb.fr/en/cephdb/), or were assumed to be equal if information was unavailable. The novel markers 16GT and 21AC were amplified in-house and amplified products were run on the Applied Biosystems 3730 DNA Analyser at the Ramaciotti Centre, University of New South Wales. We generated allele frequencies for these markers from a panel of 24 unrelated European ancestry healthy controls.

Whole-exome sequencing was performed on one unaffected and four affected Aus-12 family members, using 100-bp paired-end sequencing on the Illumina HiSeq2000 with 40× coverage, by Macrogen (Seoul, Korea).

## Results

### Clinical description of the Aus-12 family

**Fig. 1:**
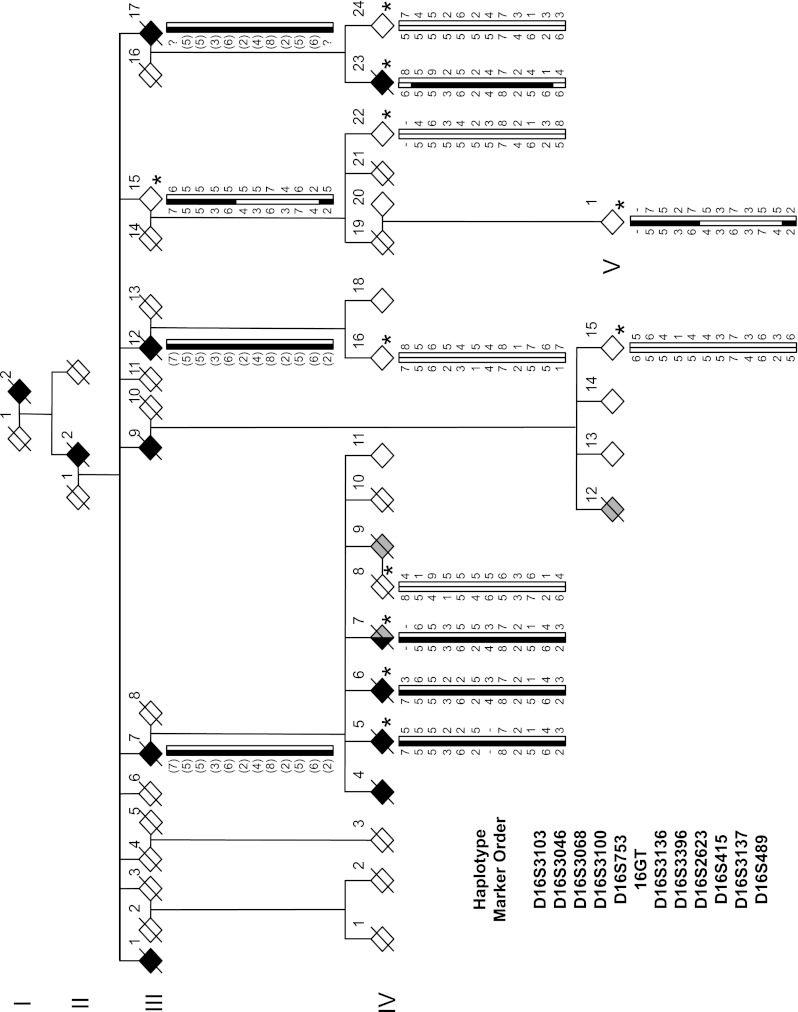
Haplotype analysis of the chromosome 16p12.3–16q12.2 region in family Aus-12. *Black symbols* show individuals with dementia, either AD or FTD; *grey symbols* individuals with ALS. A *diagonal line* marks deceased subjects. Individuals with DNA available are *asterisked*. Inferred haplotypes are in *parentheses*. Allele data for 12 markers in this region are shown. Some haplotypes were presented for review but have been omitted from publication, to protect confidentiality of at-risk individuals

Family members of pedigree Aus-12 (Fig. 1) were the subjects of detailed clinical review (Table 1). The proband (IV:23) presented with memory impairments and was given a final clinical diagnosis of FTD. The proband’s mother, III:17, died at 61 with dementia; four of her nine siblings were affected with dementia and died before age 65. The proband’s grandmother and great-grandmother were affected with dementia, dying at 64 and 40, respectively. Four first cousins of the proband had presenile dementia; two died with ALS aged 45 and 49 years, respectively; and one was diagnosed with FTD-ALS. Detailed clinical notes for pedigree members IV:23, IV:5, IV:7 and IV:12 are available in Supplementary Material.

**Table 1:** Clinical summary of family Aus-12

Subject	Age at onset (years)	Age at death (years)	Diagnosis/clinical features
I:2	30	40	Died after 10 years in psychiatric hospital
II:2	56	64	‘Presenile Alzheimer’s disease’
III:1	53	59	‘Inherited dementia’
III:7	49	56	Unspecified presenile dementia
III:9	?	64	Unspecified presenile dementia
III:12	?	62	Memory loss, apathy, lacked insight
III:17	51	61	Memory loss, wandering, behavioural problems
IV:4	56	68	Memory loss, behavioural problems
IV:5	54	64	‘FTD with aphasia’
IV:6	58	67	Unspecified presenile dementia
IV:7	62	69	FTD-ALS, Paget’s disease, parkinsonism
IV:9	?	45	Probable ALS
IV:12	?	49	ALS
IV:23	56	68	FTD

### Neuropathological analysis of Aus-12

**Fig. 2:**
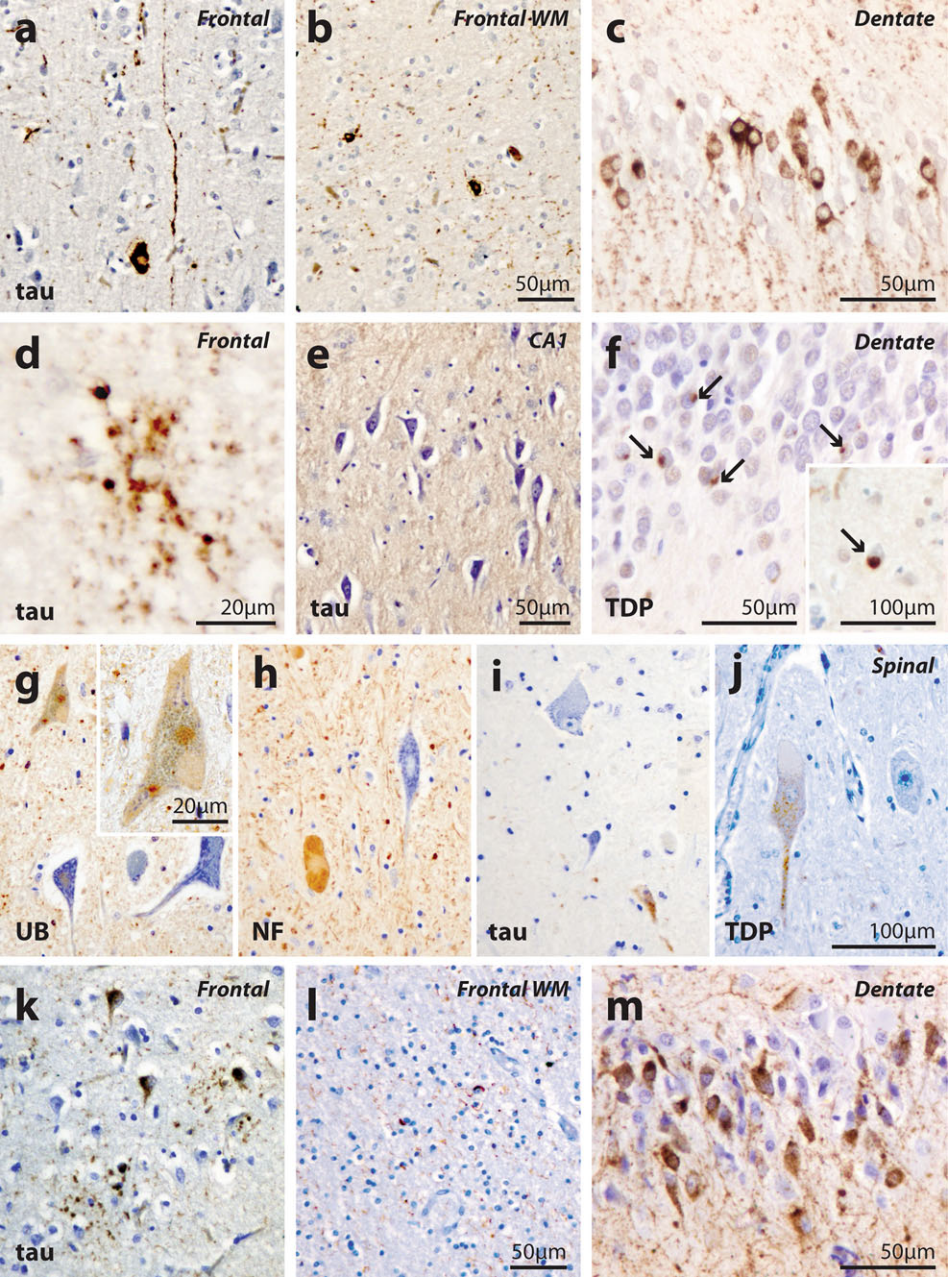
Phospho-tau and phospho-TDP neuropathology in case IV:5 (**a**–**f**), case IV:7 (**g**–**j**) and an independent CBD FTLD-tau case (**k**–**m**). Numerous phospho-tau-immunopositive threads in the frontal cortex (**a**) and white matter (**b**). **c** Dentate granule cells showing extensive phospho-tau immunoreactivity. **d** Phospho-tau-immunoreactive astrocytic plaque in frontal cortex. **e** Nissl staining showing limited loss of CA1 hippocampal pyramidal neurons. Phospho-TDP-immunopositive NCIs (*arrows*) in the dentate gyrus (**f**) and frontal cortex (**f**, *inset*). Ubiquitin- (**g**) and phosphorylated 200kD neurofilament- (**h**) immunopositive motor neurons, NCI and neurites in the cervical spinal cord. Rare phospho-tau immunoreactivity was observed in neurites, glia and small spinal cord neurons (**i**) and some phospho-TDP-immunopositive granules were observed in spinal motor neurons in the absence of TDP-positive inclusions (**j**). In the case of sporadic CBD (**k**–**m**), phospho-tau-immunoreactive astrocytic plaques, neurons and threads were seen in the frontal cortex (**k**), phospho-tau-immunoreactive glia and threads in the white matter (**l**), and diffuse cytoplasmic phospho-tau immunoreactivity was observed in the dentate granule cells (**m**)

*Case IV:5* The brain weighed 1,003 g. Microscopy confirmed severe fronto-temporal atrophy with diffuse neuronal loss and reactive gliosis maximal in the temporal poles where status spongiosis was present (Supplementary Table 2). The main immunocytochemical pathology was that of a tauopathy with the phospho-tau (AT8) immunostains showing prominent cytoplasmic neuronal reactivity (“pre-tangles”) and occasional neurofibrillary tangles involving subsets of hippocampal CA1 pyramidal neurons, temporal neocortex, frontal and parietal cortex in association with numerous AT8 immunoreactive neuronal threads (Fig. 2a–c). There was limited overall loss of hippocampal CA1 neurons (Fig. 2e). Many of the dentate granule cells showed phospho-tau cytoplasmic immunoreactivity (Fig. 2c). Occasional phosphorylated neurofilament and tau immunopositive ‘ballooned neurons’ were noted in the frontal and hippocampal sections but this was a rare finding. Phospho-tau immunoreactive astrocytic plaques (Fig. 2d) and coiled fibres were also present. A prominent subset of neurons in the nucleus basalis of Meynert showed AT8 immunoreactive cytoplasmic staining in association with numerous positive neuronal threads. Subsets of neurons and astrocytes in the caudate nucleus showed positive AT8 cytoplasmic immunostaining, whereas in the putamen and globus pallidus astrocyte immunoreactivity was more prominent than neuronal staining. Phospho-TDP-43 immunoreactive NCIs were found in both superficial and deep cortical laminae of the frontal (Fig. 2f, inset) and temporal cortex. A subset of the dentate granule cells showed phospho-TDP-43 immunopositive NCIs (Fig. 2f). Double labelling immunofluorescence revealed that cytoplasmic phospho-tau immunoreactivity and phospho-TDP-43 immunopositive NCIs were found together in approximately 20 % of hippocampal dentate granule cells and 5 % of cortical neurons (Supplementary Fig. 1c, f, j, k). While many neurons and glia contained AT8 immunoreactivity (Supplementary Fig. 1a, d, g), a minority of phospho-TDP-43 immunopositive NCIs were found in neurons that were not immunoreactive for phospho-tau (Supplementary Fig. 1c, k). No FUS or α-internexin immunoreactive inclusion pathology was observed. In the midbrain there was neuronal loss, depigmentation and gliosis of the substantia nigra. A subset of surviving substantia nigra neurons showed cytoplasmic AT8 immunoreactivity, as did scattered periaqueductal neurons in association with immunoreactive neurites. The AT8 immunostains also showed intracytoplasmic staining in neurons of the locus coeruleus, scattered neurons of midline raphe nuclei, dorsal motor vagal nuclei, nuclei ambiguii and reticular formation in association with immunoreactive neurites. The findings are those of CBD FTLD-tau associated with type B FTLD-TDP without significant bulbar motor neuron involvement.

*Case IV:7* The brain weighed 1,160 g. There was more severe frontotemporal atrophy in this case, and the substantia nigra was severely depigmented. Microscopy showed a similar pattern and type of neuropathological features to those described above but with greater neuronal loss (Supplementary Table 2), consistent with more advanced CBD FTLD-tau and type B FTLD-TDP. Western blot analysis of the sarkosyl-insoluble fraction of frontal pole tissue revealed that the insoluble tau species present in this case was consistent with a 4-repeat tauopathy, as seen with other CBD cases [5, 34] (Supplementary Fig. 2). The spinal cord was also available in this case and segmental sections showed preservation of the corticospinal tracts (myelin stain). There was patchy loss of anterior horn cells in the cervical and thoracic spinal cord segments and ubiquitinated, neurofilament-immunoreactive motor neurons, neurites (Fig. 2g, h) and NCI (Fig. 2g, inset) were observed in these sections, along with rare phospho-tau immunoreactivity (Fig. 2i). Phospho-TDP immunoreactivity was restricted to neurites and granular staining in small neurons (Fig. 2j) and absent in anterior horn cells. No FUS or α-internexin immunoreactivity was observed in these spinal cord sections.

The phospho-tau neuropathology of Aus-12 cases closely resembled that in the CBD FTLD-tau cases examined. The TDP-43 neuropathology was similar in amount and distribution but not structural type to that observed in the *GRN* mutation-positive FTLD-TDP case. Greater deposition in the dentate gyrus and fewer phospho-TDP-positive neurites was observed in the Aus-12 cases (Supplementary Material). The motor neuron involvement was mild compared with classic end-stage ALS cases and was without TDP or FUS deposition, although ubiquitinated inclusions were present (as has been observed in other familial ALS cases negative for known mutations) [23].

### Genetic analysis of Aus-12

DNA from IV:5, IV:6 and IV:24 was subjected to DNA sequence analysis of the coding regions and flanking intronic sequences for known dementia and ALS genes (Supplementary Table 1). No mutations were detected.

We undertook a genome-wide linkage analysis on 12 pedigree members, five of whom were classed as affected. Six regions generated two-point LOD scores ≥1, on chromosomes 3 (D3S1285, LOD = 1.3), 12 (D12S352, 1.0), 15 (D15S117, 1.5; D15S127, 1.1), 16 (D16S415, 1.2) and 20 (D20S107, 1.4); however, with multipoint analysis the only regions with a LOD score ≥1 were on chromosome 16 (D16S415, LOD = 2.7; D16S516, 1.1). Examination of haplotypes revealed that affected individual IV-23 was identical by state rather than by descent at D16S516: this individual harboured the same haplotype as unaffected sibling IV-24 at D16S516 and flanking markers D16S515 and D16S3091 (data not shown). In contrast, alleles at D16S415 and upstream markers D16S3046, D16S3068 and D16S3136 were shared by all affected individuals. This region on chromosome 16 was therefore fine mapped with 21 additional markers. Supplementary Table 3 details two-point LOD scores, showing a maximum of 2.4 (*θ* = 0) at D16S3396. Gene-dropping simulation analyses indicated that two chromosome 16 microsatellite markers exceeded the genome-wide 1 % significance threshold for 100 simulations. Multipoint analysis with MERLIN yielded a maximum LOD score of 2.9 for all markers from D16S753 to D16S2623 inclusive and excluded linkage (LOD < −2) at other FTD loci (*CHMP2B*, *C9ORF72*, *VCP*, *GRN*/*MAPT*) (Supplementary Fig. 3).

**Fig. 3:**
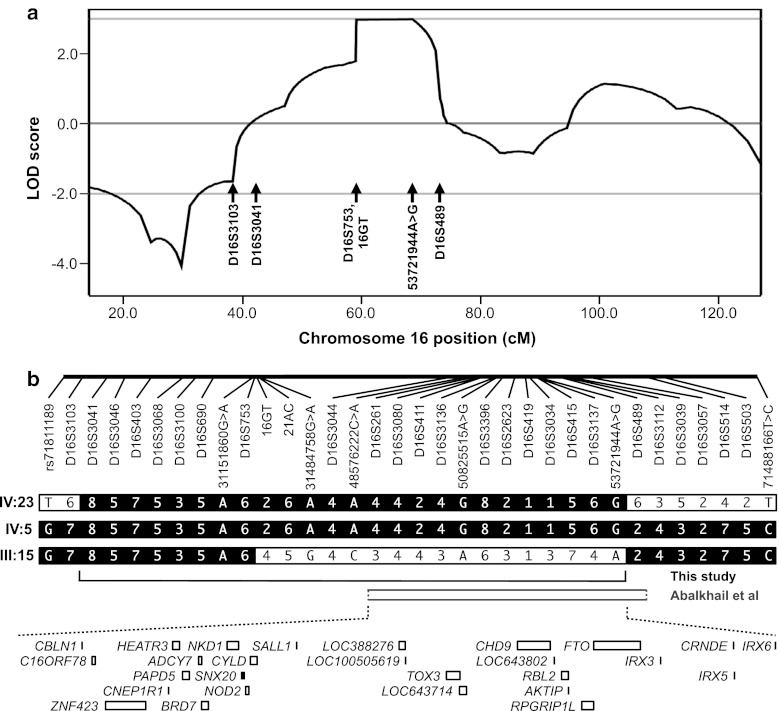
Linkage analysis of Aus-12. **a** Multipoint linkage analysis of chromosome 16, generated using MERLIN program. Positions of markers flanking critical recombinations in Aus-12 family members are indicated. **b** Haplotype analysis of chromosome 16p13.11–16q22.2. *Top* Physical positions of microsatellite markers and single-nucleotide variants used for haplotype analysis. *Middle* Haplotypes of affected individuals IV:23 and IV:5 and elderly unaffected individual III:15. The disease haplotype is indicated in *black*. The maximal critical region defined in this study and the critical region detected in the ALS family reported in Abalkhail et al. [1] are depicted below. *Bottom* RefSeq genes present in the minimal critical region, defined by overlap of Aus-12 and Abalkhail et al. critical regions

Haplotypes were constructed for the 16p12.1–q12.2 region in Aus-12 (Fig. 1). Recombinations in this family defined a 37.9-Mb region flanked by the markers D16S3103 and D16S489, in which a common haplotype was shared by all affected individuals (Fig. 3b). A smaller suggestive disease haplotype spanning 24.4 Mb was defined by recombination at D16S753 in elderly unaffected individual III:15. This region overlaps with a separate locus on 16q12.1–q12.2 reported in an ALS family by Abalkhail et al. [1] (Fig. 3b).

We performed whole-exome sequencing of affected individuals IV:5, IV:6, IV:17, IV:23 and elderly unaffected individual III:15. No pathogenic variants were identified in known dementia or ALS genes, including *FUS* on chromosome 16 (Supplementary Table 1). We identified seven variants from the exome sequencing data within the Aus-12 maximal critical region that were absent from dbSNP131, and were detected in at least 2 affected individuals and resequenced them in all 13 Aus-12 DNA samples. Four variants were present in all affected individuals and absent in the elderly unaffected individual, two of which were also absent from public databases of normal human variation (Table 2). One of these variants, g.48576222C>A, was detected in 2/934 healthy Australian individuals of European ancestry by examination of exome sequencing data (Dr Paul Leo and Prof Matthew Brown, personal communication). The second variant, g.50825515A>G, leads to the substitution of methionine to valine at amino acid position 719 of the cylindromatosis protein, CYLD. This variant is present within the region of overlap with the chromosome 16q12.1—linked ALS pedigree [1] (Table 2).

**Table 2:** Variants detected by whole-exome sequencing

gDNA change^a^	Gene	Variant type	Segregates with disease?^b^	MAF 1000G^c^	MA count EVS^d^	MAF used for linkage analysis
g.31151860G>A	*PRSS36*	Missense (p.P707L)	No	(RNC)	18/8,548	0.002
g.31447539G>A	*ZNF843*	Missense (p.P211L)	No	0	0/3,182	0.001
g.31484758G>A	*TGFB1I1*	Intronic (c.IVS1-4G>A)	Yes	0.0118	109/8600	0.012
g.48576222C>A	*N4BP1*	3′UTR (c.2691*593C>A)	Yes	0	(RNC)	0.001
**g.50825515A>G**	***CYLD***	**Missense (p.M719V)**	**Yes**	**0**	**0/8,138**	**0.001**
**g.53653005G>C**	***RPGRIP1L***	**Missense (p.A1183G)**	**No**	**0.0176**	**132/8,600**	**0.016**
**g.53721944A>G**	***RPGRIP1L***	**Intronic (c.IVS4-67A>G)**	**Yes**	**0.0059**	**(RNC)**	**0.0059**
g.71488166T>C^e^	*ZNF23*	Intronic (c.IVS3-45T>C)	No	0.0059	(RNC)	0.0059

*MA* minor allele, *MAF* minor allele frequency, *RNC* region not covered

^a^Variants present in region of overlap with a chromosome 16q12.1—linked ALS pedigree [1] are in bold. Variant nomenclature is according to the recommendations of the Human Genome Variation Society (http://www.hgvs.org/mutnomen/). Genomic co-ordinates on chromosome 16 refer to the human reference sequence GRCh37/hg19

^b^Presence in all affected individuals and absence in elderly unaffected individuals

^c^Frequency in CEU population of the 1,000 Genomes dataset (http://www.ncbi.nlm.nih.gov/variation/tools/1000genomes/)

^d^Count in European American population of the Exome Variant Server (http://evs.gs.washington.edu/EVS/)

^e^Variant lies outside Aus-12 maximal critical region

We used nine rare variants detected by whole-exome sequencing and confirmed by Sanger sequencing to repeat the linkage analysis. This generated a maximum two-point LOD score of 2.7 for markers g.31484758G>A, g.48576222C>A, g.50825515A>G and g.53721944A>G (Supplementary Table 3) and a maximum multipoint LOD score of 3.0 for markers 16GT through to g.53721944A>G inclusive (Fig. 3a).

## Discussion

In this study, we describe a complex FTD-ALS family with co-existing CBD and TDP neuropathology. The clinical presentations in this family are heterogeneous. The majority of the affected individuals presented initially with AD-like symptoms and/or motor disorders, rather than the typical presentation of FTD. For example, the proband (IV:23) presented with AD-like symptoms, but soon after developed significant personality and behavioural change, with disinhibition, socially inappropriate and obsessional behaviour, and a lack of insight and was diagnosed with FTD. Early memory impairment suggestive of AD is also observed amongst *GRN* mutation carriers [18, 30], sometimes in association with motor disorders. Patient IV:7, who was initially diagnosed with Paget’s disease, later developed clinical symptoms consistent with parkinsonism and ALS. ALS was also present as a pure syndrome in two other family members. In *MAPT* mutation carriers, ALS tends to be a late manifestation in patients already afflicted with parkinsonism, rather than a separate entity [20, 37]. Thus, clinical heterogeneity is not uncommon and has been described in other families with *GRN* and *MAPT* mutations.

The Aus-12 pedigree represents the first to our knowledge in which affected individuals have neuropathologically confirmed CBD FTLD-tau as well as type B FTLD-TDP. Concomitant phospho-tau and phospho-TDP neuropathology is not rare and has now been reported in many different sporadic neurodegenerative disorders, but particularly in AD and CBD [36]. Interestingly, although ALS is usually characterised by TDP-43 pathology [4, 28], studies have demonstrated significant deposition of phospho-tau in brains of ALS patients with cognitive impairment [14, 38]. Our western blot analyses (Supplementary Fig. 2) clearly show that the insoluble tau species comprise solely four repeat isoforms. This finding corresponds to a pathological diagnosis of CBD or PSP, rather than Pick’s disease [11]. To further differentiate between these two highly related disorders would require the presence of balloon neurons (Fig. 2) and astrocytic plaques for CBD, or globose tangles and tufted astrocytes for PSP, or perhaps the identification of a low molecular weight tau species that co-migrates with CBD and not PSP protein species [11]. The underlying phospho-TDP neuropathology of IV:5 and IV:7 was consistent with type B TDP-43 neuropathology, a pathology described in most familial FTD-ALS cases arising from *C9ORF72* mutation [12, 25]. However, the spinal cord TDP pathology was more limited, characterised by the occasional staining of neurites, with the ubiquitinated motor neuron inclusions not immunoreactive for TDP or tau. Co-immunofluorescence analysis revealed considerable overlap between the brain regions affected by the phospho-tau and phospho-TDP pathology, although both pathologies occurred independently, and more phospho-tau was observed in these cases. While the neuropathological depositions appear unique in family Aus-12, the relationship between the pathologies observed remains unclear.

Genome-wide linkage analysis resulted in the identification of a disease locus on chromosome 16p12.1–q12.2. The region overlaps with a known ALS/FTD gene, *FUS* (OMIM *137070). However, we did not detect any *FUS* mutations in Aus-12, using both Sanger sequencing and whole-exome sequencing. Individuals IV:17 and IV:23 are heterozygous for SNP rs741810 in *FUS* exon 3 and IV:5 and IV:6 are heterozygous for rs1052352 in exon 4, indicating that both alleles are present at this locus. In addition, the smaller suggestive region defined by recombination in individual III:15 does not include the *FUS* locus. Intriguingly, the Aus-12 disease region overlaps with that identified in an independent ALS family (Fig. 3b), with maximal multipoint LOD scores of 2.06 at D16S3080 and D16S411 [1]. This ALS family is negative for *FUS* mutations and the hexanucleotide repeat expansion in *C9ORF72* (Prof Jacqueline de Belleroche, personal communication). Assuming that the disease in this family is due to the same genetic cause, the recombination breakpoint observed in this study at D16S489 slightly narrows the telomeric boundary of the combined published minimal disease region by 26 kb. This combined critical region contains 27 RefSeq genes (Fig. 3b). We detected one nonsynonymous variant within these genes by whole-exome sequencing: Met719Val in *CYLD*. *CYLD* was previously sequenced in affected individuals from the Abalkhail et al. [1] ALS pedigree but no mutations were identified (Prof Jacqueline de Belleroche, personal communication). CYLD Met-719 is invariant in mammals, chicken, frog and zebrafish, but is valine in *Drosophila* CYLD homologues (data not shown). In silico analysis using programs Align GVGD (http://agvgd.iarc.fr/agvgd_input.php/), PolyPhen-2 (http://genetics.bwh.harvard.edu/pph2/) and SIFT (http://sift.jcvi.org/) predicted that this substitution was not likely to be pathogenic. This implies that the disease variant is either located in non-exonic sequence or is of a type that cannot be detected by direct sequencing, such as a genomic rearrangement, copy-number variant, or a repeat expansion.

The identification of two independent families with linkage to the same locus indicates that this region may harbour a second major FTD-ALS gene. The positional cloning of the major FTD causative loci have been instrumental in the elucidation of pathogenic mechanisms underlying the various neuropathological variants, including *MAPT* and *GRN* [6, 16]. Genetic evaluation of family Aus-12 will further aid our understanding of disease pathogenesis in FTD-ALS cases with both phospho-tau and phospho-TDP deposition.

## Electronic supplementary material

Below is the link to the electronic supplementary material.

Supplementary material 1 (PDF 7947 kb)
