# Supplementary material for: Frontotemporal dementia–amyotrophic lateral sclerosis syndrome locus on chromosome 16p12.1–q12.2: genetic, clinical and neuropathological analysis
Source: Acta Neuropathol. 2013 Jan 22;125(4):523–33. doi: 10.1007/s00401-013-1078-9 (PMC3611035; doi:10.1007/s00401-013-1078-9)
Supplement: Supplementary file 1 — Supplementary material 1 (PDF 7947 kb) [file 401_2013_1078_MOESM1_ESM.pdf]

SUPPLEMENTARY MATERIAL

Frontotemporal dementia–amyotrophic lateral sclerosis syndrome locus on chromosome 16p12.1-q12.2

Carol Dobson-Stone*, Agnes A. Luty*, Elizabeth M. Thompson, Peter Blumbergs, William S. Brooks, Cathy L. Short, Colin D. Field, Peter K. Panegyres, Jane Hecker, Jennifer A. Solski, Ian P. Blair, Janice M. Fullerton, Glenda M. Halliday, Peter R. Schofield, and John B. J. Kwok

*Equal first authors

C. Dobson-Stone, A. A. Luty, W. S. Brooks, J. M. Fullerton, G. M. Halliday, P. R. Schofield, J. B. J. Kwok: Neuroscience Research Australia, Sydney, Australia; and University of New South Wales, Sydney, Australia

E. M. Thompson: SA Clinical Genetics Service, SA Pathology, Women's and Children's Hospital, Adelaide, Australia; and Department of Paediatrics, University of Adelaide, North Terrace, Adelaide, Australia

P. Blumbergs: Institute of Medical and Veterinary Science, Adelaide, Australia

C. L. Short: Department of Neurology, The Queen Elizabeth Hospital, Woodville, Australia

C. D. Field: formerly at Memory Clinic, Division of Rehabilitation & Aged Care, Repatriation General Hospital, Daw Park, Australia; now at Adelaide Dementia Driving Clinic, North Adelaide, Australia

P. K. Panegyres: Neurodegenerative Disorders Research Pty Ltd, Subiaco, Australia

J. Hecker: Department of General Medicine, Royal Adelaide Hospital, Adelaide, Australia

J. A. Solski, I. B. Blair: Northcott Neuroscience Laboratory, ANZAC Research Institute, Concord Hospital, Sydney, Australia

CORRESPONDING AUTHOR: Dr John B. J. Kwok, Neuroscience Research Australia, Barker St, Randwick, NSW 2031, Australia

Phone: +61 2 9399 1025
Fax: +61 2 9399 1005
E-mail: j.kwok@neura.edu.au

Supplementary Materials and Methods

Western blot analysis of tau isofoms

Sarkosyl-insoluble fractions were isolated from 250 mg frontal cortex of affected Aus-12 individual IV:7, one neuropathologically confirmed CBD case (4-repeat tau dominant tauopathy) and two neuropathologically confirmed AD cases (3- and 4-repeat tauopathy) according to the protocol described by de Calignon et al [39]. Approximately 15 to 30 micrograms of total protein were electrophoresed using SDS-PAGE gels. Following transfer of proteins onto nitrocellulose membrane (Bio-Rad Laboratories, Hemel Hempstead, UK), monoclonal antibodies against either 3-repeat tau (clone 8E6/C11 at 1:2000 dilution, Merck Millipore, Kilsyth, Australia) or 4-repeat tau (clone 1E1/A6 at 1:500 dilution, Merck Millipore) were used to determine the dominant type of sarkosyl-insoluble tau species. Immunoblotting with a monoclonal antibody against β-actin (clone C4, Merck Millipore) was used to visualise the level of total protein per lane. Protein bands were visualised using enhanced chemiluminescence according to manufacturer's instructions (Supersignal West Pico Chemiluminescent Substrate, Thermo Scientific, Rockford, USA).

Clinical notes for Aus-12 family

Case IV:23 - The proband, IV:23, was referred to a specialist geriatrician at age 56 because over the previous year she had begun to repeat questions, misplace items and become lost when driving. She would forget the names of friends and would fail to recognise familiar faces. She was unable to manage her previous tasks of helping with the accounts and with managing horses. She was less fastidious about her personal appearance than before. Initially she felt she was deteriorating like her mother, but at the time of referral she denied having problems. Friends were concerned about her driving. She had become verbally aggressive at times and made inappropriate comments, which was a change from her previous personality. Despite this, she scored 27/30 on the Mini-Mental State Examination (MMSE), losing 2 points for recall and 1 for copying intersecting pentagons. On a 10-word learning task she was able to recall 5/10 after an interval. There were some difficulties with higher intellectual function including description of word similarities and differences and rather concrete proverb definition, but she had reasonable verbal fluency. Calculation was impaired. Drawings and constructional tasks were initially well preserved, and it was noted that she was easily able to copy complex figures or spontaneously sketch faces and figures. Prominent early memory symptoms and geographical disorientation, together with the strong family history of clinically diagnosed Alzheimer's disease (AD), led to an initial clinical suspicion of AD. However after a year she developed significant personality and behavioural change, with disinhibition, socially inappropriate and obsessional behaviour, and a lack of insight. This, together with continuing intact constructional skills and left frontotemporal hypoperfusion on a SPECT scan, led to a diagnosis of FTD. Her condition gradually progressed. She required residential care from age 66 for management of her behavioural and self-care deficits. Interestingly her drawing skills remained one of the few areas of preservation, implying relative preservation of parietal function. She died at age 68 years.

Case IV:5 - This former forklift driver moved interstate and lived alone after a divorce. His behaviour became increasingly odd from his early to mid-fifties. A brain CT scan when he was 59 was reported as normal. His house was squalid and unsafe, with the rooms filled with garbage. There was no running water or electricity as he had disconnected the wires. He was admitted to a psychiatric institution at 63, by which time he was largely mute. He hoarded the belongings of other residents and displayed challenging, repetitive and impulsive behaviour. Some extrapyramidal features noted at the time were thought to be related to previous treatment with haloperidol. Brain CT was reported as showing temporal lobe atrophy. The clinical diagnosis was FTD. He became less mobile, developed swallowing difficulties and pneumonia and died at age 64 years. Autopsy revealed bilateral bronchopneumonia.

***Case IV:7* -** This former welder had not worked since being retrenched at age 54 years. He had Paget's disease of both hips as well as ischaemic heart disease. His wife reported that he had given up many of his hobbies and interests from age 50. After a coronary bypass operation at 62 he developed progressive problems with memory, orientation and language. When seen by a neurologist at 67 he had become withdrawn and lacked emotion. There was some evidence of disinhibited behaviour. He had parkinsonian features including expressionless face, increased tone and shuffling gait together with pout and grasp reflexes and some utilisation behaviour. He had some fluctuating alertness but no hallucinations. Mini-Mental State score was 12/30 and he scored 0/18 on the Frontal Assessment Battery [41]. The initial clinical diagnosis was FTD with parkinsonism, however by age 68 he had developed widespread fasciculations. Electromyography detected evidence of active denervation in three limbs, with fibrillation and sharp wave potentials and/or fasciculations, thus confirming ALS according to Awaji criteria

[40]. The parkinsonian features continued, however, and were treated with L-dopa and carbidopa, with improvement in his mobility. He developed pneumonia and died aged 69 years. Autopsy revealed bilateral bronchopneumonia.

Case IV:12 **–** The limited clinical notes available for this patient indicated that he presented with flaccid paralysis, dysarthria and dysphagia. His tongue exhibited wasting and fasciculations, there was severe muscle wasting in the upper limbs, and he exhibited a positive jaw jerk. The sensory examination was normal. These observations are consistent with a clinical diagnosis of ALS. According to family record, he died at age 49 years.

Pathological comparison of Aus-12 cases with familial FTLD-TDP and sporadic CBD FTLD-Tau

As previously described [29], the familial FTLD-TDP case with a *GRN* mutation had typical type A FTLD-TDP neuropathology with numerous phospho-TDP-immunoreactive NCIs, glial cytoplasmic inclusions, short dystrophic neurites, and the occasional neuronal intranuclear inclusion. No pathological FUS or α-internexin immunoreactivity was observed in this case. Occasional phospho-tau and neurofilament immunoreactivity was observed in the entorhinal cortex, consistent with age-associated changes. In contrast, the two CBD FTLD-tau cases had phospho-tau-immunopositive astrocytic plaques and coiled bodies as well as phospho-tau immunopositive threads and neurons (Fig. 2k-m). Ballooned neurons were also immunopositive for phosphorylated neurofilament and to a lesser extent phospho-tau, as previously described [10]. No pathological FUS or α-internexin immunoreactivity was observed in these cases, and only occasional granules of abnormal phospho-TDP were observed.

The phospho-tau neuropathology of Aus-12 closely resembled the CBD FTLD-tau cases. The most similar features were the considerable dentate involvement (Fig. 2c,m) and the very prominent glial phospho-tau immunoreactivity in the white matter. The TDP-43 neuropathology on the other hand was similar in amount and distribution but not structural type to the familial FTLD-TDP case with a *GRN* mutation, which had phospho-TDP-immunoreactive NCI and neurites in the cortical regions examined. However, greater phospho-TDP deposition occurred in the dentate gyrus and fewer phospho-TDP-immunopositive neurites were found in family Aus-12 (Fig. 2f) compared to the *GRN* mutation case.

Supplementary references

39. de Calignon A, Polydoro M, Suárez-Calvet M, William C, Adamowicz DH, Kopeikina KJ, Pitstick R, Sahara N, Ashe KH, Carlson GA, Spires-Jones TL, Hyman BT (2012) Propagation of tau pathology in a model of early Alzheimer's disease. Neuron. 73:685-697.

40. de Carvalho M, Dengler R, Eisen A, England JD, Kaji R, Kimura J, Mills K, Mitsumoto H, Nodera H, Shefner J, Swash M (2008) Electrodiagnostic criteria for diagnosis of ALS. Clin Neurophysiol 119:497-503

41. Dubois B, Slachevsky A, Litvan I, Pillon B (2000) The FAB: a Frontal Assessment Battery at bedside. Neurology 55:1621-1626

42. Ott J (1991) Analysis of human genetic linkage. Johns Hopkins University Press, Baltimore.

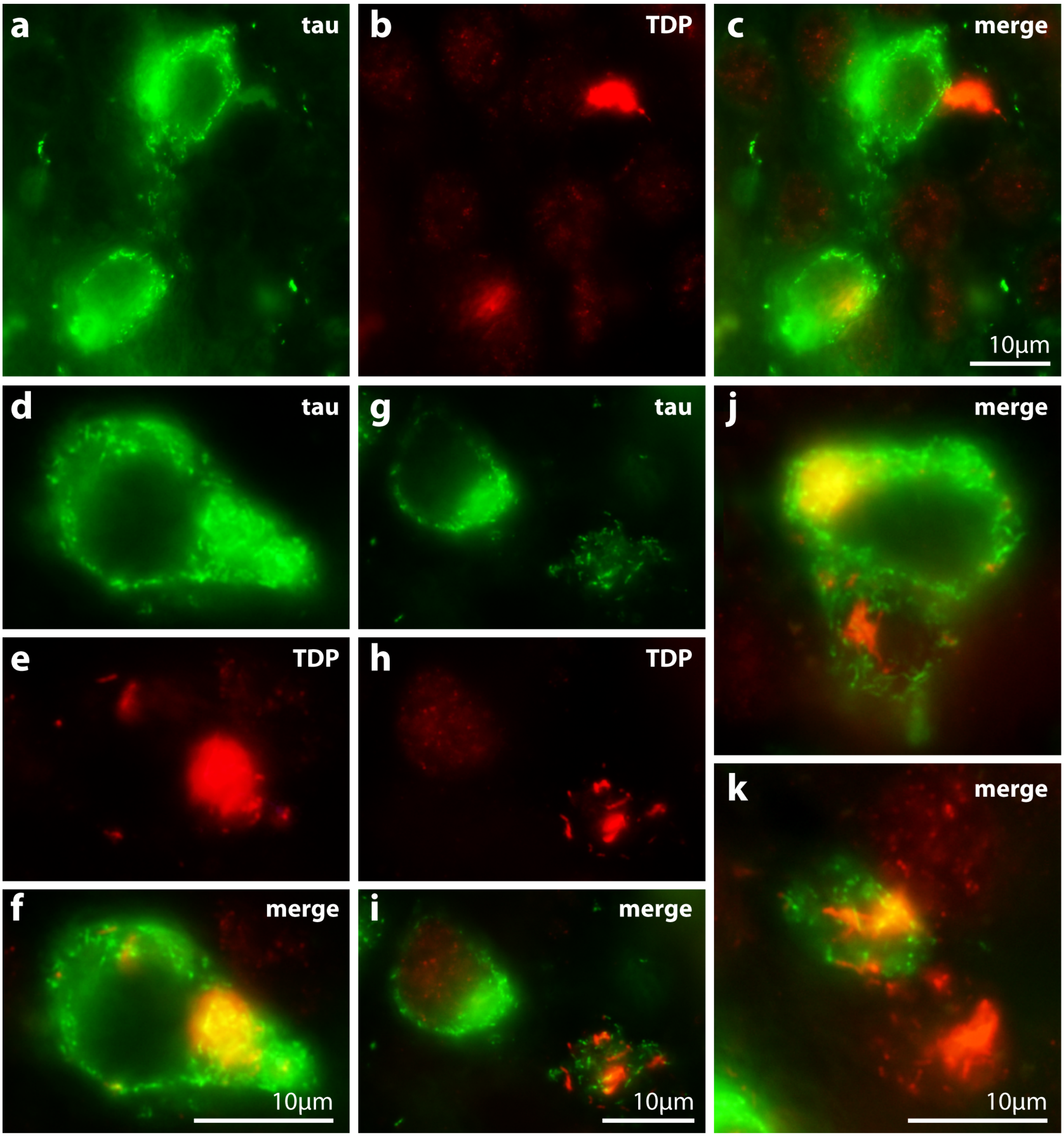

Supplementary Fig. 1 Double label immunofluorescence of the dentate granule cells in case IV:5 with phospho-tau (green) and phospho-TDP (red). Merged images of neurons in panels **a-c**, **d-f**, **g-i**, **j** and **k** show overlapping (yellow) and separate phospho-tau (green) and phospho-TDP (red) pathologies. **i** immunoreactivity of a fibrillar inclusion

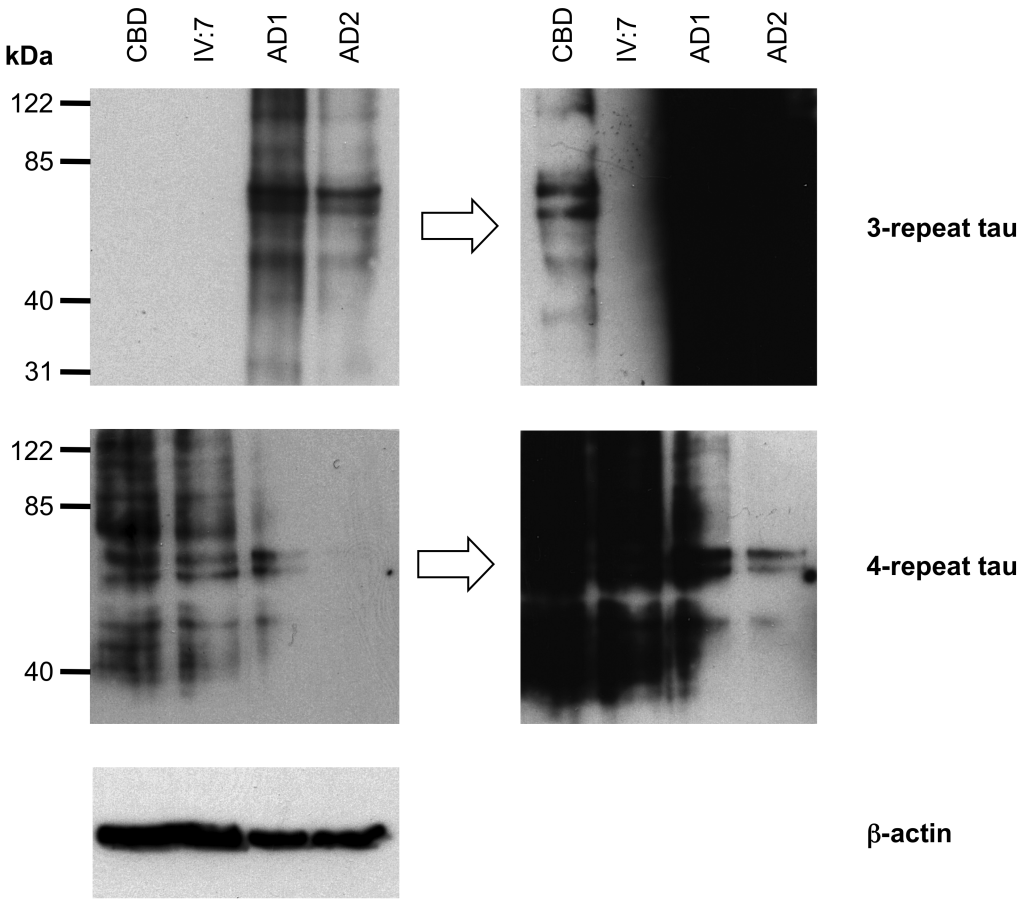

Supplementary Fig. 2: Western blot analysis of microtubule-associated protein tau isoforms from sarkosyl-insoluble fraction of brain lysates. Monoclonal antibodies against specific splice isoforms of tau were used to determine the type of tauopathy in Aus-12 individual IV:7, with a corticobasal degeneration (CBD) case and two Alzheimer's disease (AD) cases as comparison. A long exposure of each autoradiograph (right panels) is included to identify proteins present at low levels. Note that IV:7 has no detectable 3-repeat tau.

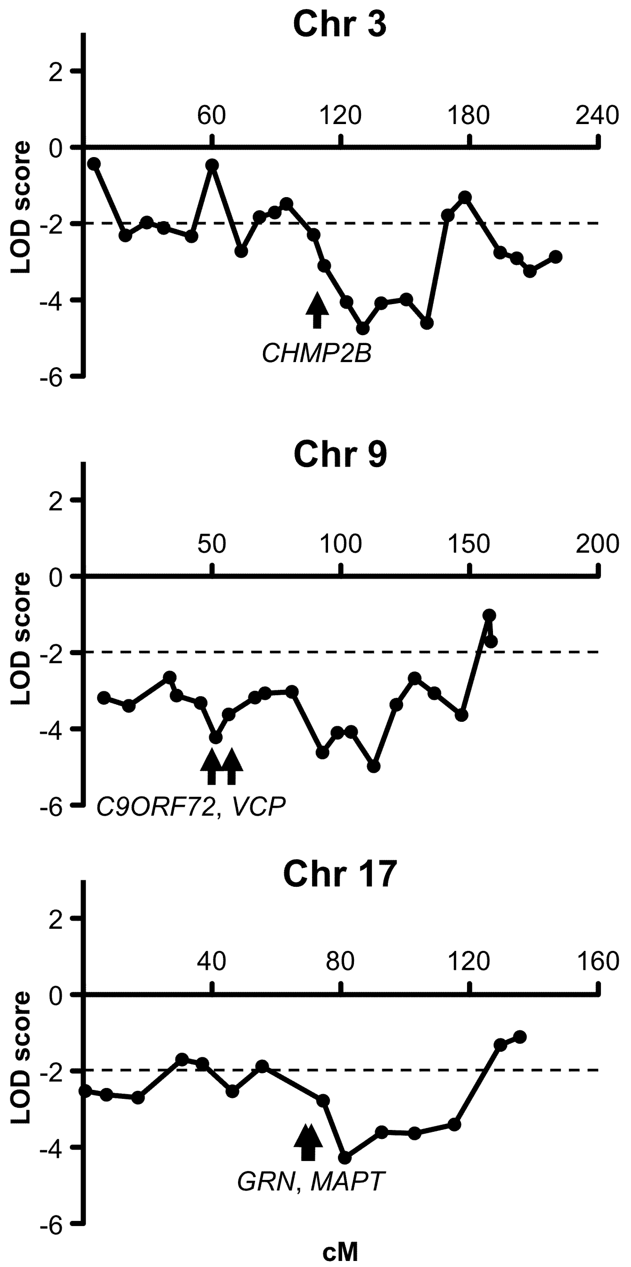

Supplementary Fig. 3: Exclusion of Aus-12 linkage to known FTD loci. Graphs depict multipoint LOD scores for chromosomes 3, 9 & 17. The dashed line indicates LOD of -2: chromosomal regions below this are statistically excluded as disease loci for this family [42]. Positions of known FTD genes *CHMP2B*, *C9ORF72*, *VCP*, *GRN*, *MAPT* are arrowed.

Supplementary Table 1 Known dementia/ALS genes screened for mutations in Aus-12

Gene	Sanger sequencing[a]	Whole-exome sequencing
APP	+	+
C9ORF72[b]	-	+
CHMP2B	+	+
FUS	+	+
GBA	-	+
GRN	+	+
LRRK2	-	+
MAPT	+	+
OPTN	-	+
PARK2	-	+
PARK7	-	+
PINK1	-	+
PRNP	-	+
PSEN1	+	+
PSEN2	+	+
SNCA	-	+
SNCB	-	+
SOD1	+	+
TAF15	-	+
TARDBP	+	+
UBQLN1	-	+
UBQLN2	-	+
VCP	+	+

[a]Direct DNA sequencing of the coding regions and 50 base pairs of flanking intronic sequences was performed in one unaffected and two affected individuals, using BigDye v3.1 chemistry (Applied Biosystems, CA, USA). Primer sequences and PCR conditions available on request. Sequencing reactions were run on the Applied Biosystems 3730 DNA Analyser at the Ramaciotti Centre, University of New South Wales, Australia.

[b]All available family members were screened for the *C9ORF72* repeat expansion, using techniques described previously [12].

Supplementary Table 2

Cases	Pathological feature *	Region #					
		Frontal cortex	Temporal cortex	Hippocampus	Caudate/putamen	Substantia nigra	Cerebellum
Case IV:5	Neuronal loss	+ - ++	0 - +++	0 - +	0	++	0
	Neuronal pathology†	tau and TDP43	tau and TDP43	tau and TDP43	tau	tau	-
	Glial tau pathology	++ - +++	+ - +++	+++	++ - +++	+++	+
Case IV:7	Neuronal loss	++	++	+ - ++	0	+++	0
	Neuronal pathology†	tau and TDP43	tau and TDP43	tau and TDP43	tau	tau	-
	Glial tau pathology	+++	+++	++	++	+++	0
CBD FTLD-tau cases N=2	Neuronal loss	+-++	0 - +	0 - +	0 - +++	++ - +++	0
	Neuronal pathology†	tau	tau	tau	tau	tau	-
	Glial tau pathology	+++	++	++	+++	+++	0 - +
GRN mutation FTLD-TDP case	Neuronal loss	+++	+	++	0 - +	+++	0
	Neuronal pathology†	TDP43	TDP43	TDP43	TDP-43	-	-
	Glial tau pathology	0	0	0	0	0	0

* 0, +, ++, +++ = none, mild, moderate, severe † presence of neuronal tau and/or TDP-43 pathology

# Regions incorporate a number of different Brodmann areas, eg. for frontal cortex frontal association, limbic and motor cortices were all sampled; for temporal cortices superior and inferior, anterior and posterior regions were sampled; anterior and posterior levels of the hippocampus were sampled and the different sectors and gyri included in the analysis; both the caudate nucleus and putamen were evaluated.

Supplementary Table 3 Two-point LOD scores[a] for Aus-12 on chromosome 16p13.2-q12.2

Locus	Chr 16 location[c]	θ =				
		.0	.1	.2	.3	.4
D16S404	9,718,112	-0.98	-0.04	0.04	0.04	0.02
D16S3075	12,209,198	-3.79	-0.74	-0.28	-0.08	-0.01
D16S3103	17,473,463	-2.41	-0.75	-0.35	-0.15	-0.04
D16S3041	*19,388,757*	*-0.09*	*0.01*	*0.01*	*-0.02*	*-0.02*
D16S3046	*20,886,398*	*-0.23*	*-0.16*	*-0.10*	*-0.05*	*-0.01*
D16S403	*23,037,651*	*0.71*	*0.62*	*0.47*	*0.27*	*0.07*
D16S3068	*25,560,601*	*0.15*	*0.15*	*0.11*	*0.05*	*0.01*
D16S3100	*26,581,626*	*1.39*	*1.24*	*0.94*	*0.58*	*0.20*
D16S690	*27,958,387*	*0.38*	*0.43*	*0.35*	*0.20*	*0.05*
31151860G>A	*31,151,860*	*1.45*	*1.43*	*1.14*	*0.75*	*0.29*
D16S753	*31,273,449*	*0.34*	*0.34*	*0.28*	*0.17*	*0.05*
16GT[b]	***31,437,877***	***1.97***	***1.50***	***1.01***	***0.52***	***0.14***
31447539G>A	***31,447,539***	***-0.10***	***-0.09***	***-0.06***	***-0.03***	***-0.01***
21AC[b]	***31,466,316***	***1.64***	***1.18***	***0.72***	***0.34***	***0.09***
31484758G>A	***31,484,758***	***2.68***	***2.16***	***1.60***	***0.98***	***0.34***
D16S3044	***47,437,536***	***-0.02***	***-0.01***	***-0.00***	***-0.00***	***-0.00***
48576222C>A	***48,576,222***	***2.74***	***2.23***	***1.66***	***1.03***	***0.37***
D16S261	***49,238,067***	***0.70***	***0.55***	***0.36***	***0.18***	***0.04***
D16S3080	***49,683,350***	***0.72***	***0.73***	***0.59***	***0.37***	***0.12***
D16S411	***49,736,734***	***0.24***	***0.26***	***0.21***	***0.12***	***0.03***
D16S3136	***50,706,233***	***0.63***	***0.48***	***0.30***	***0.13***	***0.02***
50825515A>G	***50,825,515***	***2.74***	***2.23***	***1.66***	***1.03***	***0.37***
D16S3396	***51,192,308***	***2.43***	***1.93***	***1.38***	***0.79***	***0.24***
D16S2623	***52,109,704***	***1.35***	***1.12***	***0.78***	***0.41***	***0.11***
D16S419	***52,953,362***	***0.72***	***0.58***	***0.40***	***0.21***	***0.06***
D16S3034	***53,140,502***	***-0.11***	***-0.02***	***0.02***	***0.03***	***0.02***
53653005G>C	***53,653,005***	***-0.10***	***-0.09***	***-0.06***	***-0.03***	***-0.01***

D16S415	***53,670,661***	***1.36***	***1.17***	***0.84***	***0.46***	***0.13***
D16S3137	***53,682,437***	***1.23***	***1.07***	***0.80***	***0.46***	***0.14***
53721944A>G	***53,721,944***	***2.71***	***2.20***	***1.63***	***1.00***	***0.36***
D16S489	55,359,315	-0.23	0.74	0.66	0.44	0.16
D16S3112	55,670,551	-0.89	0.15	0.24	0.17	0.06
D16S3039	55,999,944	-1.35	-0.23	-0.06	-0.02	-0.01

Markers within the disease haplotype are in italics; markers within suggestive disease haplotype defined by recombination in elderly unaffected III:15 are in bold.

[a]LOD scores were obtained by use of FASTLINK v4.1P (http://www.ncbi.nlm.nih.gov/CBBresearch/Schaffer/fastlink.html)

[b]Novel microsatellite markers analysed in this study: primer sequences and PCR conditions are available on request.

[c]Genomic co-ordinates on chromosome 16 refer to the human reference sequence GRCh37/hg19. For microsatellite markers, the 5' position of the upstream primer is indicated.
